# Analyzing *In Silico* the Relationship Between the Activation of the Edema Factor and Its Interaction With Calmodulin

**DOI:** 10.3389/fmolb.2020.586544

**Published:** 2020-12-04

**Authors:** Irène Pitard, Damien Monet, Pierre L. Goossens, Arnaud Blondel, Thérèse E. Malliavin

**Affiliations:** ^1^Unité de Bioinformatique Structurale, Institut Pasteur and CNRS UMR 3528, Paris, France; ^2^Center de Bioinformatique, Biostatistique et Biologie Intégrative, Institut Pasteur and CNRS USR 3756, Paris, France; ^3^Ecole Doctorale Université Paris Sorbonne, Paris, France; ^4^Institut Pasteur, rue du Dr Roux, Unité Yersinia, Paris, France

**Keywords:** protein-protein interaction, *Bacillus anthracis*, virulence factor, Cavity detection, allostery

## Abstract

Molecular dynamics (MD) simulations have been recorded on the complex between the edema factor (EF) of *Bacilllus anthracis* and calmodulin (CaM), starting from a structure with the orthosteric inhibitor adefovir bound in the EF catalytic site. The starting structure has been destabilized by alternately suppressing different co-factors, such as adefovir ligand or ions, revealing several long-distance correlations between the conformation of CaM, the geometry of the CaM/EF interface, the enzymatic site and the overall organization of the complex. An allosteric communication between CaM/EF interface and the EF catalytic site, highlighted by these correlations, was confirmed by several bioinformatics approaches from the literature. A network of hydrogen bonds and stacking interactions extending from the helix V of of CaM, and the residues of the switches A, B and C, and connecting to catalytic site residues, is a plausible candidate for the mediation of allosteric communication. The greatest variability in volume between the different MD conditions was also found for cavities present at the EF/CaM interface and in the EF catalytic site. The similarity between the predictions from literature and the volume variability might introduce the volume variability as new descriptor of allostery.

## Introduction

As the interactions between proteins are essential in all biological processes, the modulation of these interactions with small ligands is a promising direction (Morelli et al., 2011; Tuffery and Derreumaux, 2012; Huang, 2014; Zhang et al., 2014; Aguirre et al., 2015; Kuenemann et al., 2015; Fischer et al., 2016; Shin et al., 2017). During the last decade, virtual screening has experienced a turning point where interest has widened from protein active sites to cryptic sites (Beglov et al., 2018; Vajda et al., 2018) not visible in the isolated protein conformation but formed upon ligand binding. These cryptic sites have been proposed to be detected by analysis of protein structures (Kozakov et al., 2015; Cimermancic et al., 2016), by mixed-solvent MD simulations (Ghanakota et al., 2019; Martinez-Rosell et al., 2020) or by biased MD simulations (Comitani and Gervasio, 2018; Sun et al., 2020). Inhibition of protein-protein interactions, search for new cryptic sites and targeting protein function in an allosteric way are three closely related goals. Targeting allostery (Tschammer, 2016; Deredge et al., 2017; Feng et al., 2018; Ni et al., 2019) is particularly suitable in the case of inhibition of protein-protein interactions, because the conformational changes in which allosteric communication plays an important role when establishing the interaction, may create or modify cavities (Goodey and Benkovic, 2008).

Allostery, discovered in the early days of molecular biology (Monod et al., 1965; Koshland et al., 1966) has since then, evolved from a model of discrete protein conformations to a more continuous description of the free energy landscape of proteins (Goodey and Benkovic, 2008; Liu and Nussinov, 2016; Wodak et al., 2019). Consequently, the allostery is nowadays quite often described as being closely connected to the variations of equilibrium between protein conformations. Networks of protein residues have been pointed out to be involved in the transmission of these equilibrium variations (Gur et al., 2013; van den Bedem et al., 2013; Raman et al., 2016). The evolution of allostery description has allowed the emergence of so-called allosteric ligands (Goodey and Benkovic, 2008; Nussinov and Tsai, 2013), for which the binding to the protein has a long-range influence on the protein conformation via residue networks. In this regard, a pioneering approach has been based on a detailed mechanistic simulation of functional motions (Laine et al., 2010a). The allosteric ligands and the associated allosteric pockets attract a lot of attention, because they expand the range of pockets and ligands that could be exploited in effector design by virtual screening studies (Zhang and Nussinov, 2019). In addition, the diversity of allosteric phenomena can be utilized to limit the emergence of resistance mutations in target proteins, making carefully selected allosteric ligands more robust to the appearance of resistance in pathogens.

As a result, numerous methods have been developed for allosteric pocket detection in protein structures (Panjkovich and Daura, 2014; Xu et al., 2018; Ghode et al., 2020; Tan et al., 2020). In particular, it was shown (Ma et al., 2016) that most of the known allosteric site motions show high correlations with corresponding orthosteric site motions, whereas other surface cavities did not. Many of the prediction methods are based on a description of protein structures as an elastic network in which each residue is replaced by the atom Cα and the structure deformations are modeled through a set of springs between these atoms (Panjkovich and Daura, 2014; Guarnera and Berezovsky, 2019). The structure-based statistical mechanical model of allostery (SBSMMA) (Guarnera and Berezovsky, 2019) has been introduced which permits to calculate the free energy variation due to allosteric communication.

**Figure 1:**
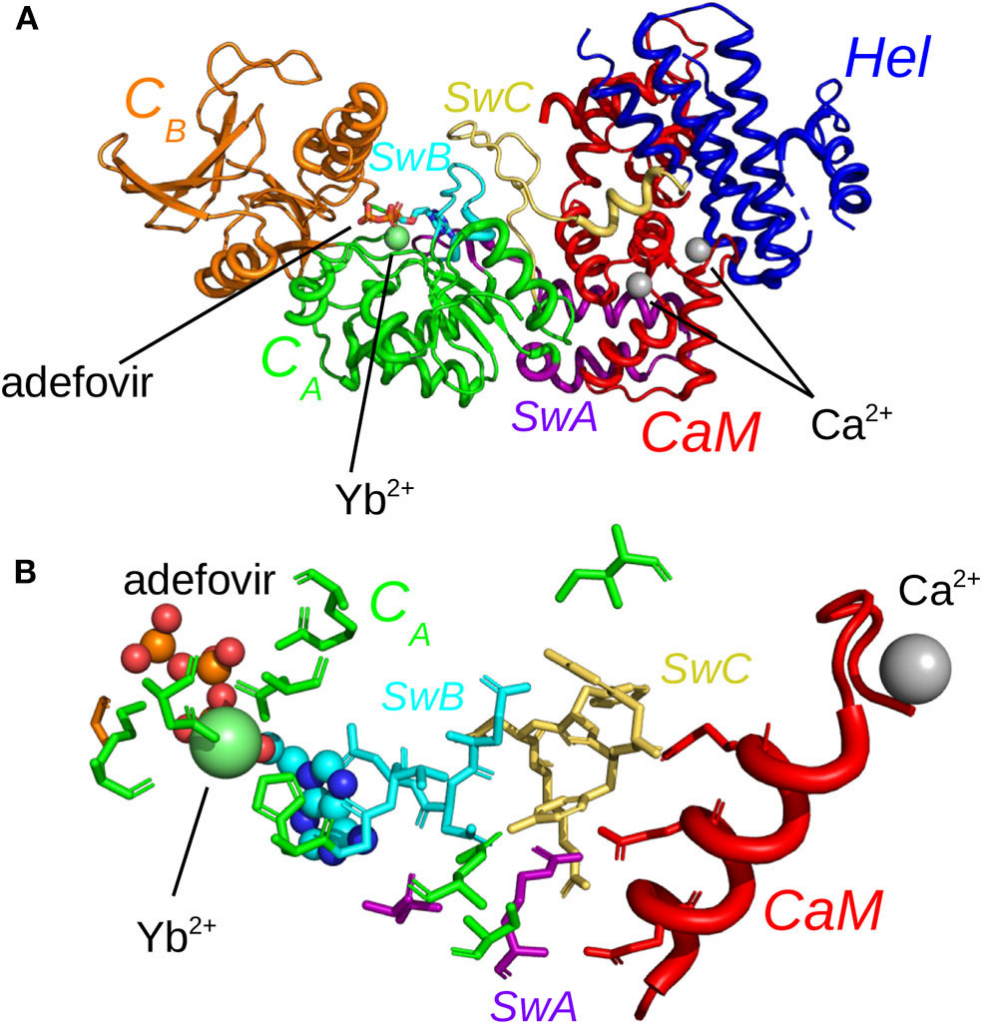
**(A)** X-ray crystallographic structure (PDB entry: 1PK0) of the complex EF/CaM with the ions Ca^2+^ colored in gray and the ion Yb^2+^ colored in lime. The ligand adefovir is drawn in ball-and-sticks, the regions C_*A*_ (residues 292-349 and 490-622), C_*B*_ (residues 350-489) and helical (residues 660-800, labeled “Hel” on the figure). of EF are colored in green, orange and blue and CaM is colored in red. The switches A (residues 502-551), B (residues 578-591), and C (residues 630-659), labeled SwA, SwB, and SwC, are colored in purple, cyan, and yellow. The helix V of CaM is colored in salmon. **(B)** Network of residues in the X-ray crystallographic structure (PDB entry: 1PK0) of the complex EF/CaM connecting residues of the catalytic site to residues of the α helix V of CaM. The ligand adefovir is drawn in spheres. The helix V is drawn in carton. The residues of CaM, region C_*A*_ and of switches A, B, and C are in the same colors than in **(A)**.

In the present work, we investigate the use of molecular dynamics (MD) simulations and bioinformatics approaches to analyze protein-protein interactions, variability of cavities and allostery for one example of protein-protein interaction corresponding to the activation of a virulence factor. The Edema Factor (EF) of *Bacillus anthracis* is activated in the cytoplasm of the host cell by interacting with the ubiquitous protein calmodulin (CaM). This interaction depends on the level of Ca^2+^ loaded by CaM, the C terminal lobe of CaM (C-CaM) displaying the highest affinity for ions Ca^2+^ during the interaction with EF (Ulmer et al., 2003). The EF/CaM complex has been extensively studied by structural biology and biophysical techniques (Drum et al., 2000, 2001, 2002; Shen et al., 2002, 2004a, 2005; Ulmer et al., 2003; Guo et al., 2004, 2008) as well as by molecular modeling (Laine et al., 2008, 2009, 2010a,b, 2012; Martínez et al., 2009). This complex (Figure 1A) represents a very good example of an interaction with induced conformational selection for both partners. Indeed, free CaM in solution displays a very heterogeneous set of conformations, with wide range of relative re-orientations of N terminal (N-CaM) and C terminal (C-CaM) lobes (Bertini et al., 2004; Anthis et al., 2011), whereas CaM in complex with EF is blocked in an extended conformation. Similarly, the inactive state and the activated state of EF display largely different conformations. The helical region (residues 660-800) is moved apart from the C^*A*^ (residues 292-349 and 490-622) region to allow CaM insertion. A large conformational reorganization of switches A (residues 502-551, purple), B (residues 578-591, cyan), and C (residues 630-659, yellow) also takes place and the catalytic site is reshaped in its active organization (Figure 1A). Strikingly, in switch C two strings β and a connection loop present in the structure of isolated EF are converted to a α helix in the structure of the EF/CaM complex.

In the literature, both orthosteric and allosteric ligands have been proposed to inhibit EF activity. Several orthosteric inhibitors, binding to the catalytic site, have been discovered (Soelaiman et al., 2003; Shen et al., 2004b; Chen et al., 2009; Taha et al., 2009, 2012; Geduhn et al., 2011). Among them, the ligand adefovir (Shen et al., 2004b) was found by X-ray crystallography to bind in the catalytic site in the presence of an Yb^3+^ ion coordinated by adefovir as well as by protein residues. On the other hand, the compound 10506-2A has been shown to be an IPPI (inhibitor of protein-protein interaction) and to bind close to the EF helical regions (Lee et al., 2004). Thiophen ureoacid ligands have been discovered following virtual screening on the pocket SABC, formed by residues from the three switches A, B, and C (Laine et al., 2010a). Since it is believed that they do not bind to the enzymatic site of EF, compounds 10506-2A and thiophene ureoacids must by definition bind to an allosteric site.

Here, we propose the following approach to detect protein regions which should be targeted using an allosteric approach to inhibit the activity of EF. Starting from the X-ray crystallographic structure of EF/CaM complex bound to the orthosteric inhibitor adefovir (Shen et al., 2004b), we destabilized it by removing alternatively several co-factors: the ion Mg^2+^ present in the catalytic site, the ions Ca^2+^ loaded by CaM or the ligand adefovir. The analysis of the trajectories made it possible to detect a network of hydrogen bonds and stacking interactions, connecting the EF catalytic site and the EF/CaM interface and showing a strong destabilization when the co-factors are removed from the EF/CaM structure. In addition, cavities present in the EF/CaM complex have been tracked along all MD trajectories, and cavity volume variability has been proposed as a method of detecting allosteric pockets. The results obtained by this approach on EF/CaM have been confirmed by analyzes (Xu et al., 2018; Guarnera and Berezovsky, 2019). Therefore, the network of hydrogen bonds and stacking interactions connecting the EF catalytic site and the EF/CaM interface could be considered a plausible candidate for the allosteric communication path within the complex structure.

## Materials and Methods

### Preparation of the Systems for MD Simulations

The X-ray crystallographic complex of EF with the inhibitor adefovir (Shen et al., 2004b) (PDB entry: 1PK0, Figure 1A) served as the starting point of the MD trajectories. The protein chain was analyzed using Molprobity (Chen et al., 2010) (molprobity.biochem.duke.edu), in order to add hydrogen atoms and to select the sidechains orientations optimizing the network of hydrogen bonds. The ion Yb^3+^ present in the catalytic site, was replaced by a physiologically compatible ion Mg^2+^.

The files to perform MD simulations were prepared with the CHARMM GUI interface (www.charmm-gui.org) (Lee et al., 2016; Jo et al., 2017). The chains A and D of the structure 1PK0 were neutralized using potassium ions and solvated with water molecules (Table 1). The loop (residues 675-695) located between helices L and M (Drum et al., 2002) in the helical domain is disordered and not visible in the initial 1PK0 structure. This missing loop was added to the structure using the CHARMM GUI interface. The force field CHARMM36 (MacKerell et al., 1998, 2004; Best et al., 2012) and the TIP3P water model (Jorgensen et al., 1983) were used to model the physical interactions. The parameters for ligand adefovir were obtained using the CHARMM GUI interface (www.charmm-gui.org). with the Ligand Reader and Modeler tool (Kim et al., 2017). Six different systems have been prepared with different molecular compositions, starting from the structure 1PK0 then by removing various co-factors: ions Mg^2+^, Ca^2+^ and adefovir (Table 1).

**Table 1:** Systems composition.

**System**	**Solute**	**Number of TIP3P waters**	**Neutralizing K^+^ ions**	**Total number of atoms**
EF_ade_Mg_CaM_Ca	EF, CaM, Mg^2+^,2 Ca^2+^, adefovir	68,219	9	215,183
EF_ade_CaM_Ca	EF, CaM, 2 Ca^2+^, adefovir	68,220	11	215,187
EF_ade_Mg_CaM	EF, CaM, Mg^2+^, adefovir	69,773	13	219,847
EF_ade_CaM	EF, CaM, adefovir	69,795	15	219,914
EF_CaM_Ca	EF, CaM, 2 Ca^2+^	69,849	11	220,034
EF_CaM	EF, CaM	69,785	15	219,844

### Recording MD Trajectories

The MD trajectories were recorded using NAMD 2.13 (Phillips et al., 2005) (www.ks.uiuc.edu/Research/namd/). The simulations were performed in the NPT ensemble. A cutoff of 12 Å and a switching distance of 10 Å were defined for non-bonded interactions, while long-range electrostatic interactions were calculated with the Particle Mesh Ewald (PME) protocol (Darden et al., 1993). The RATTLE algorithm (Ryckaert et al., 1977; Andersen, 1983) was used to keep all covalent bonds involving hydrogens rigid, enabling a time step of 2 fs. Atomic coordinates were saved every 10 picoseconds. At the beginning of each trajectory, the system was first minimized for 10,000 steps, then heated up gradually from 0 to 300 K in 300,000 integration steps. Then, the system was equilibrated for 50,000 steps. For each of six various conditions (Table 1), two independent trajectories of 200 ns were recorded (named the replicas R1 and R2) and corresponding to a total simulation duration of 2.4 μs.

### Analysis of MD Trajectories

The root-mean-square deviations (RMSD, Å) of atomic coordinates, their root-mean-square fluctuations (RMSF, Å), as well as distance and angle analysis between atoms along the recorded trajectories were performed using cpptraj (Roe and TE Cheatham, 2013). Angles between CaM α helix axes were calculated using python scripts based on the python MDAnalysis library (Michaud-Agrawal et al., 2011; Gowers et al., 2016), the helices being defined as CaM regions including residues 8-19 (helix I), 31-37 (helix II), 46-53 (helix III), 66-73 (helix IV), 83-92 (helix V), 103-110 (helix VI), 119-127 (helix VII), 139-145 (helix VIII). The axis of an helix spanning residues *n* to *p* is defined as a segment connecting the geometric centers of the atoms Cα^*n*^ and Cα^*n*+2^ and of the atoms Cα^*p*^ and Cα^*p*+2^.

The solvent accessible surfaces of residues along the trajectory were calculated using a python script based on the python MDAnalysis library (Michaud-Agrawal et al., 2011; Gowers et al., 2016) and the software FreeSASA (Mitternacht, 2016). The EF catalytic site surface was defined as the sum of solvent accessible surfaces of EF residues H351, K353, S354, K372, R329, K346, L348, D491, D493, H577, G578, T579, D582, N583, E588, F586, and T548. The surface of the SABC pocket was defined as the sum of the solvent accessible surfaces of EF residues A496, P499, I538, E539, P542, S544, S550, W552, Q553, T579, Q581, L625, Y626, Y627, N629, and N709. The surface of hydrophobic patches are defined according to Yang et al. (2004). The N-CaM patch is formed by the N-CaM residues A10, F12, A15, L18, F19, L32, M36, L39, M51, V55, M71, M72, and M76. The C-CaM patch is formed by the C-CaM residues I85, A88, V91, F92, L105, M109, L112, L116, M124, F141, M144, M145, and A147. The accessible surface was calculated using the Lee-Richards algorithm (Lee and Richards, 1971) with a probe radius of 1.4 Å.

Cavities of the EF/CaM complex were detected using the software *mkgridXf* (Monet et al., 2019). The cavities are delimited between two surfaces: the inner and out surfaces determined by rolling probes of radii 1.4 and 8 Å on the protein atoms. In addition, allosteric pockets were predicted using the Web server of the PARS approach (Panjkovich and Daura, 2014) (bioinf.uab.cat/cgi-bin/pars-cgi/pars.pl).

## Results

### The Removal of Co-factors Destabilizes the Organization of the EF/CaM Complex

**Figure 2:**
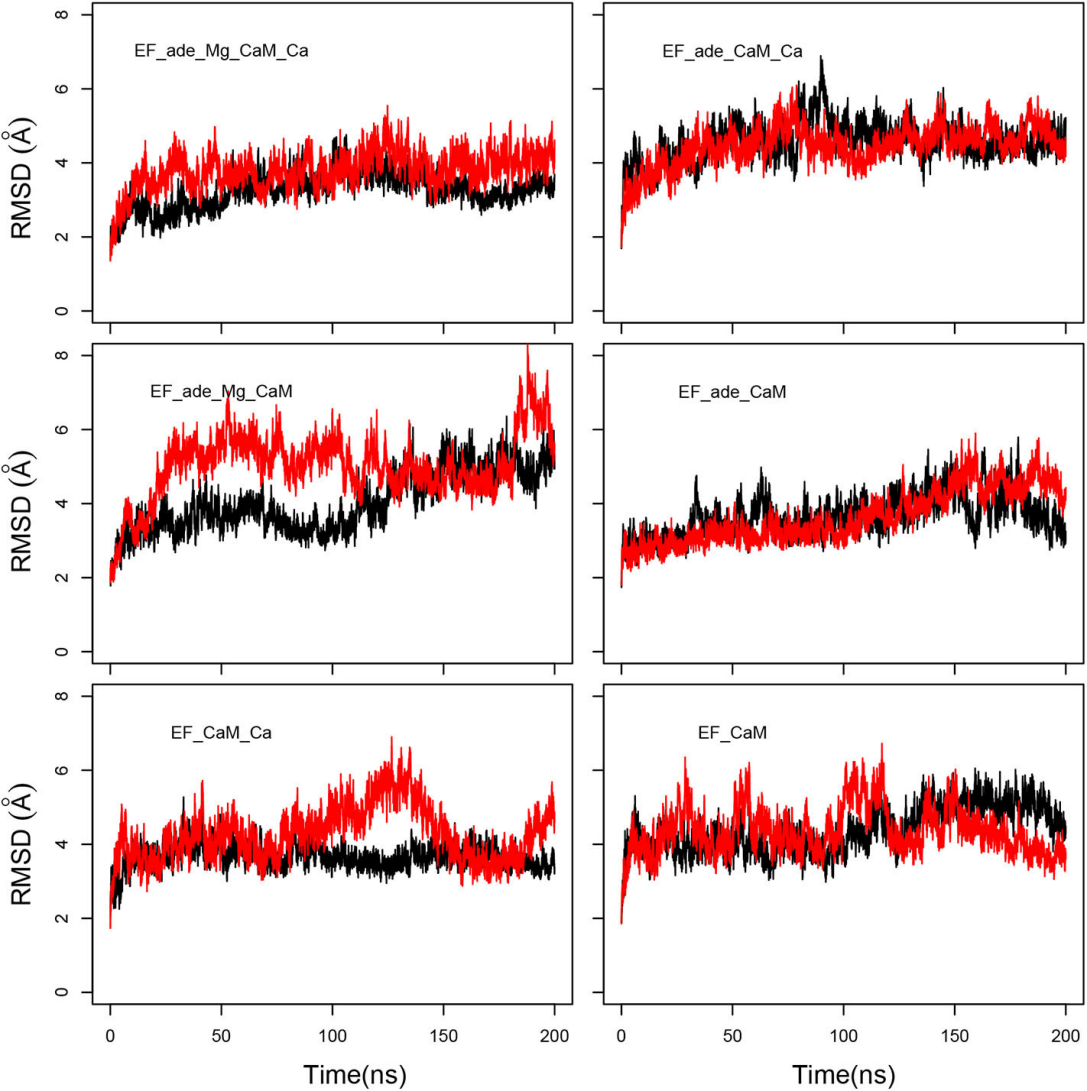
Coordinate root-mean-square deviations (RMSD: Å) of the backbone heavy atoms of EF and CaM with respect to the PDB structure 1PK0, calculated along all trajectories. For each trajectory, the black and red curves correspond to the two replicas R1 and R2.

The evolution of coordinate root-mean-square deviations (RMSD, Å) with respect to the initial conformations of chains A (EF) and D (CaM) in the structure 1PK0 calculated along the trajectories, displays quite similar trends, with a plateau attained in most of the cases, after 50 ns, and located between 4 and 6 Å (Figure 2). The reproducibility of the replicas of a given trajectory has been further analyzed by realizing a clustering on each replica. This clustering was performed using a self-organizing map approach, described in the Supplementary Material. RMSD coordinates were calculated between the representative conformations extracted from clustering by comparing them two by two (Supplementary Figure 1). The average RMSD values between replicas of the same trajectory (yellow cells) are larger than the average RMSD values within each replicas (gray cells), but are mostly smaller than the average RMSD between different trajectories. Interestingly, EF_ade_Mg_CaM_Ca, which corresponds to the full system, displays the smallest RMSD of 3.9 Å between the replicas.

**Figure 3:**
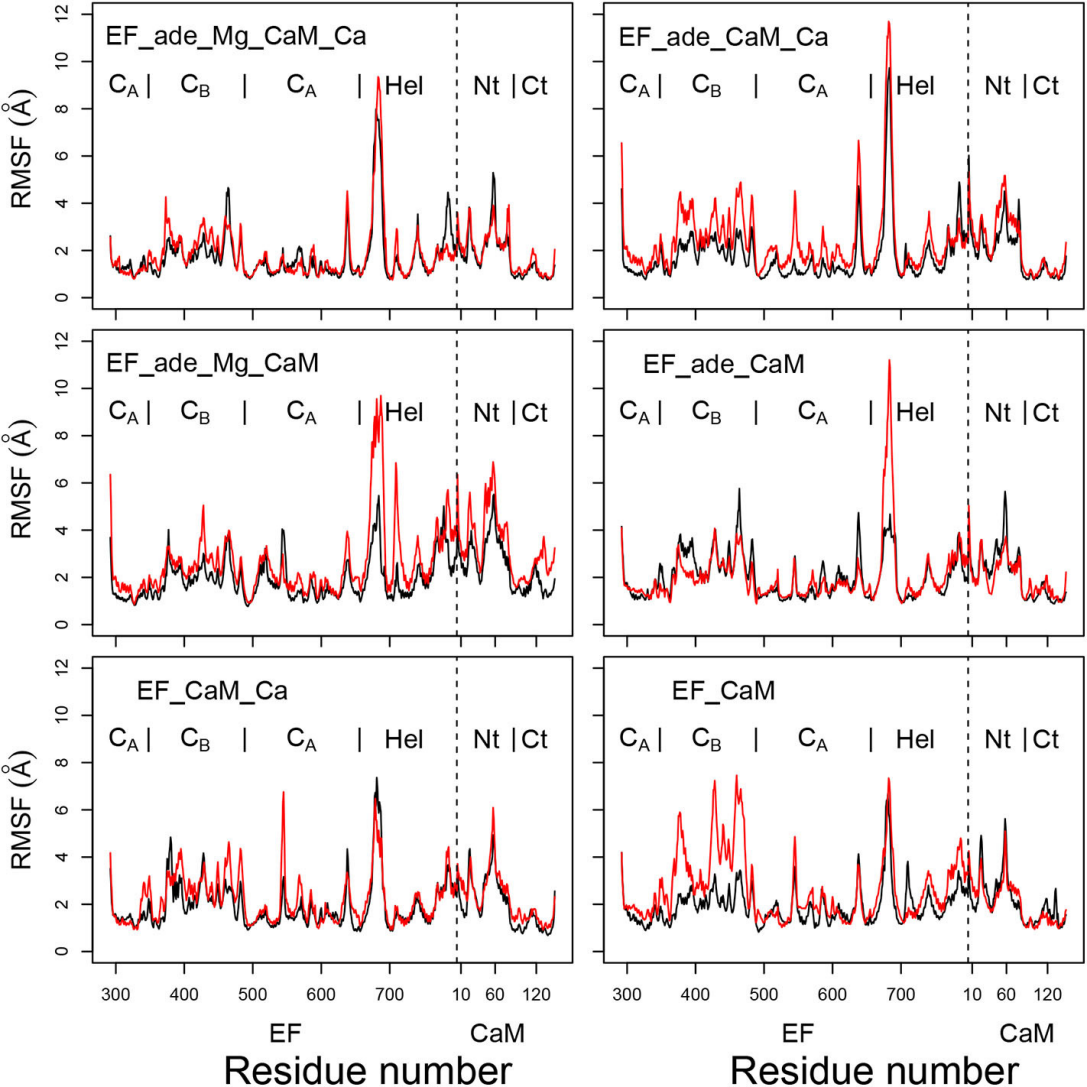
Coordinate root-mean-square fluctuations (RMSF: Å) of the backbone heavy atoms of the complex EF/CaM, each frame being superimposed on the chains A and D of the PDB entry 1PK0. For each trajectory, the black and red curves correspond to replicas R1 and R2. The N terminal residue of CaM is indicated by a vertical dashed line, the EF and CaM regions are labeled on the top of each plot. The N-CaM and C-CaM regions are labeled Nt and Ct for sake of clarity.

The coordinate root-mean-square fluctuations (RMSF, Å) (Figure 3) display similar profiles for all trajectories. Unsurprisingly, a certain variability is observed for the peak of fluctuations on the loop (residues 675-695) missing in the initial X-ray crystallographic structure and modeled when preparing the system for molecular dynamics simulation, as described in section 2. Globally, the N-CaM region fluctuates more than the C-CaM region, loaded with Ca^2+^ ions.

Interestingly, the removal of ions does not increase much the mobility provided that the ligand adefovir is still present (trajectory EF_ade_CaM). By contrast, the removal of one type of ion in the presence of the ligand (trajectories EF_ade_CaM_Ca and EF_ade_Mg_CaM) or of the ions and the ligand (trajectory EF_CaM) increases the internal mobility of EF and produces a shift between the replicas. The trajectory EF_CaM_Ca, corresponding to the active toxin ready to interact with adefovir, has an internal mobility similar to that of EF_ade_Mg_CaM_Ca. The similarity of internal mobility for these two EF/CaM complexes mirrors their functional correspondence.

**Figure 4:**
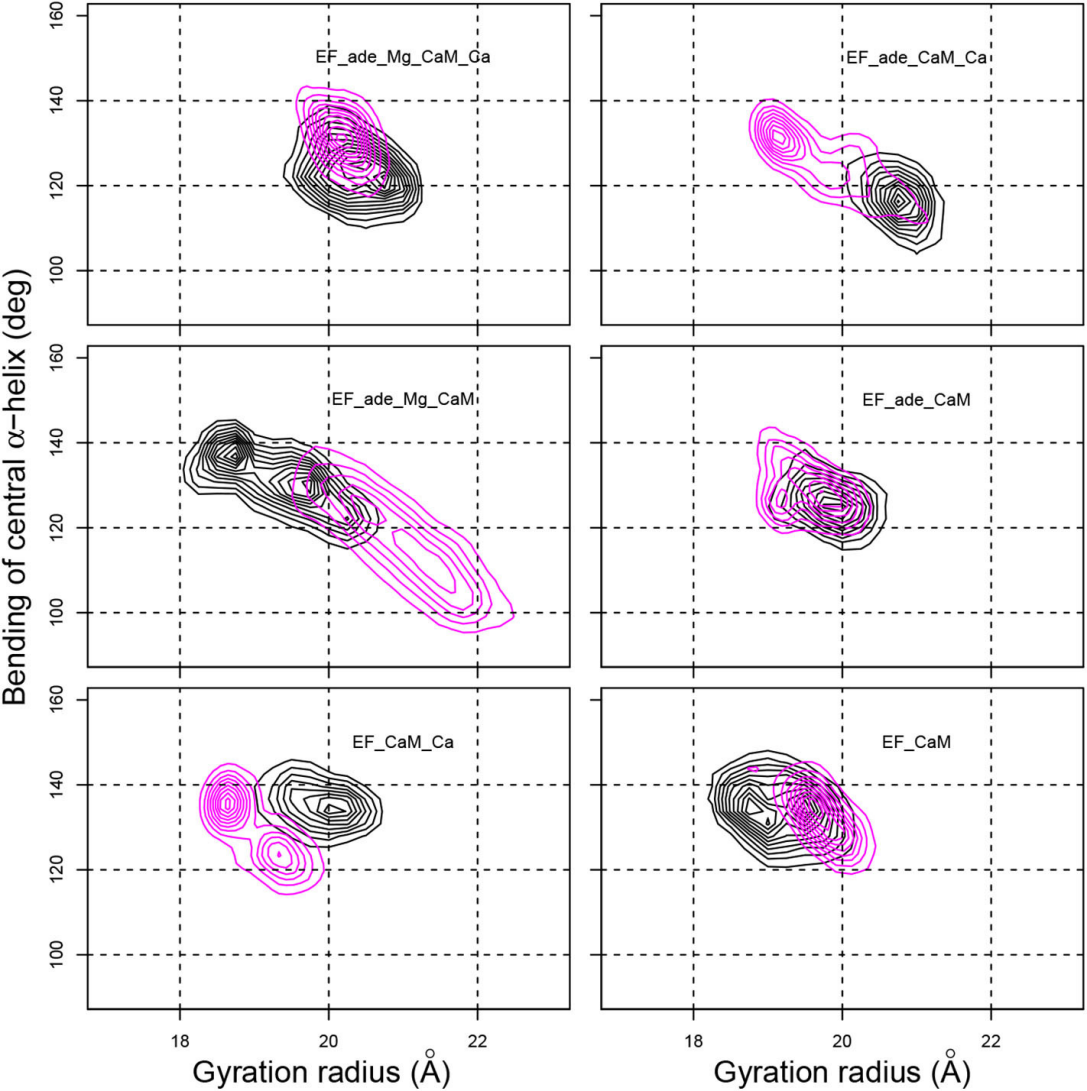
Contour plots describing the variation of the gyration radius (Å) with respect to the bending angle of the CaM central α-helix (deg) determined as the angles between axes of α helices IV (residues 66-73) and V (residues 83-92). The contour lines describe the joint probability distribution of these two parameters. For each trajectory, the black and magenta contours correspond to replicas R1 and R2.

The global shape of the EF/CaM complex was analyzed by monitoring the gyration radius of the complex as well as the bending angle of the central α helix of CaM defined as the angle between the axes of helices IV and V (Figure 4). The gyration radius mainly samples values in the 19–22 Å range, with the exception of EF_ade_Mg_CaM in which the gyration radius vary in the 18–21 Å range (Figure 4, x-axis). Removal of Magnesium (EF_ade_CaM_Ca) or of Calcium (EF_ade_Mg_CaM) ions in the presence of adefovir induces an increase in the range of variations. On the other hand, the removal of the ligand makes it possible to maintain a narrow distribution of values, but shifts the radius of gyration to smaller values, around 19 Å. Thus both ligand and ions have an influence on the complex expansion.

The bending of the central α helix of CaM, monitored as the angle between axes of α helices IV and V (Figure 4, y-axis), is relatively stable around 130° for the full system EF_ade_Mg_CaM_Ca as well as for EF_CaM_Ca and EF_CaM. But, the systems EF_ade_CaM_Ca and EF_ade_Mg_CaM, in which the adefovir is present and only one ion type is conserved show large drifts of angle toward smaller angles down to 100° or larger angles up to 140°. Furthermore, the 2D contour plot describing the joint probability distribution of the gyration radius and the bending angle (Figure 4) reveals a strong correlation between the variations of these two parameters for systems EF_ade_CaM_Ca and EF_ade_Mg_CaM.

The drifts in the bending angle of the central α helix is a sign of a destabilization of the EF/CaM complex. Indeed, CaM displays in the EF/CaM complex a relatively unusual extended conformation (Yamniuk and Vogel, 2004). It was highlighted in Laine et al. (2008) using normal mode analysis of different trajectories recorded on EF/CaM that an exact fit of the extended conformation of CaM to the EF structure is required for the complex stability. Indeed, in the EF/CaM complex, the extended structure of CaM is used to push away the helical domain from the remaining part of EF. The variation of bending angle in CaM central helix has a direct influence on the extension of the conformation of CaM and therefore on its adjustment to the activated EF. On the other hand, the variation of gyration radius of the whole complex corresponds to a major perturbation and perturbs the catalytic site and catalytic activity of EF. Thus the correlation observed between the gyration radius and the bending angle (Figure 4) proves that the fit of extended CaM to the complex EF/CaM has an influence on the EF function.

Overall, the removal of ions induces perturbations in the EF/CaM complex, which displays important conformational changes highlighted by variations of the gyration radius. These perturbations are visible through the atomic fluctuations as well as the correlated variations of gyration radius and CaM central α helix bending. The bending of the central helix α of CaM, due to the local environment, is related to the radius of gyration, describing the general shape of the complex, thus highlighting a long-distance effect.

### CaM Conformation Inside the EF/CaM Complex Conserves Features of the Isolated CaM

The CaM conformation will be analyzed and compared to the literature (Crivici and Ikura, 1995) information in order to assess the fitting of CaM to the interaction with EF. CaM is an extremely flexible protein (Bertini et al., 2004; Anthis et al., 2011), its conformation being strongly modulated by the loading of Calcium ions (Finn B.E. et al., 1995; Zhang et al., 1995; Komeiji et al., 2002). This allows CaM to bind various target proteins and peptides involved in the signaling processes (Ikura et al., 1992).

In CaM, the EF-hand domains are helix-loop-helix motifs responsible for Calcium binding. The Calcium ions bound to the C-CaM lobe, are coordinated by the carbonyl oxygen of residue Y99 and side chain carbonyl groups of residues D93, D95, and E104 in the EF-hand 3, and by the carbonyl oxygen of residue Q135 and side chain carbonyl groups of residues D131, D133, and E140 in the EF-hand 4. The average coordination distances reveal that the ions keep similar coordination geometry along all trajectories (Supplementary Table 1). Side chain atoms Oδ from residues D93, D95 and residues D131, D133 show weaker coordination than the other coordinated residues.

It is well-known from the literature that the presence of Calcium ions has a strong influence on the conformation of the isolated CaM. The angles between the α helices of EF hands in CaM increase, as well as the accessible surfaces of hydrophobic patches, upon Calcium loading (Finn B.E. et al., 1995; Zhang et al., 1995; Yamniuk and Vogel, 2004). In addition, in the absence of Calcium ions, the central α helix is more disordered (Barbato et al., 1992; Komeiji et al., 2002). These variations of CaM conformation in the presence of Calcium ions allows a better interaction of CaM with peptides involved in Calcium signaling (Ikura et al., 1992; Crivici and Ikura, 1995).

**Figure 5:**
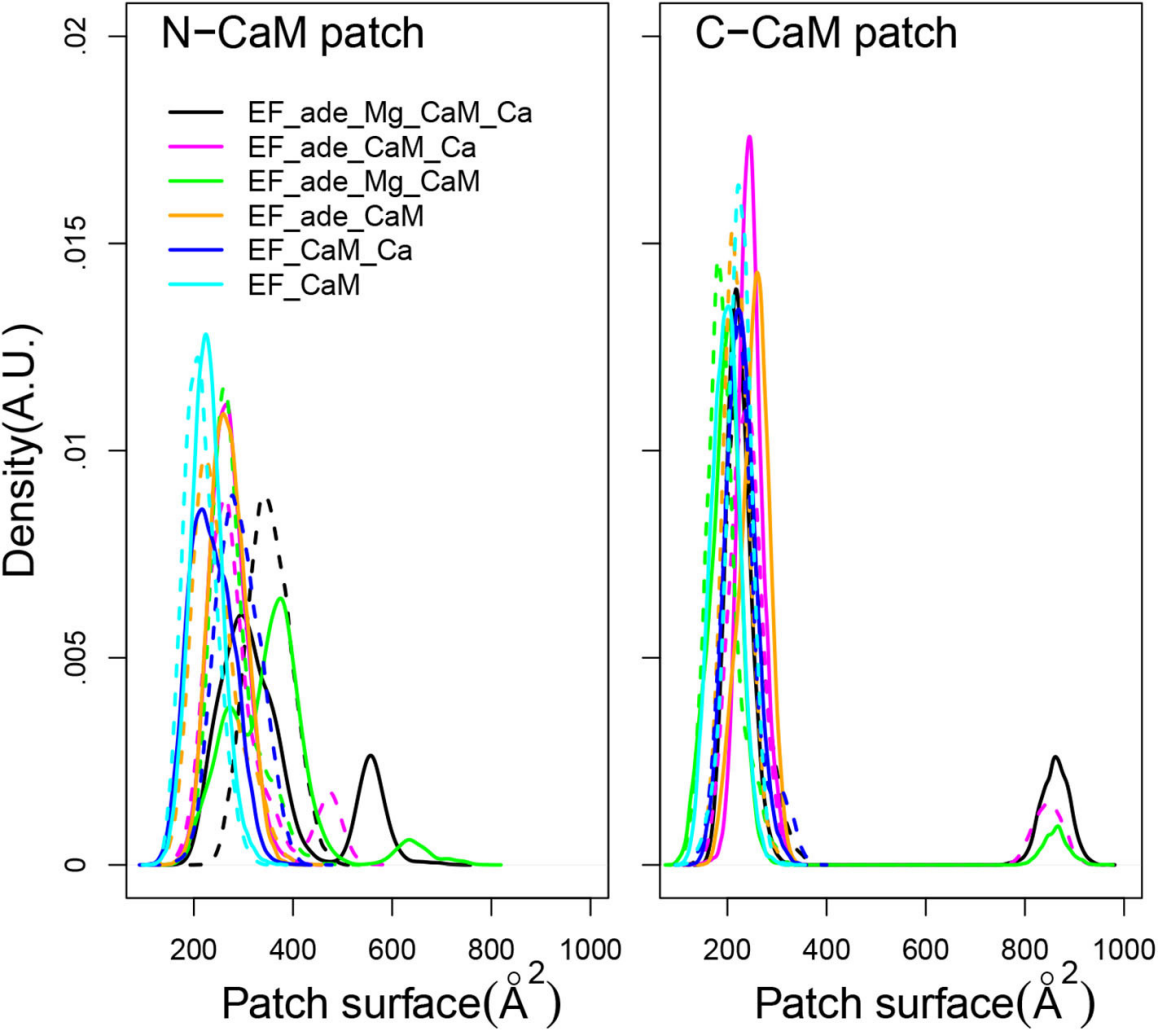
Distribution of accessible surface (Å^2^) of the hydrophobic patch of N-CaM and C-CaM. The N-CaM patch is defined by the residues A10, F12, A15, L18, F19, L32, M36, L39, M51, V55, M71, M72, M76. The C-CaM patch is defined by the residues I85, A88, V91, F92, L105, M109, L112, L116, M124, F141, M144, M145, A147. For each trajectory, the plain and dashed curves correspond to replicas R1 and R2.

Using as definition of the hydrophobic patches the residues previously listed in section 2, the surfaces of hydrophobic patches of N-CaM and C-CaM (Figure 5) display quite different trends. The C-CaM patch, corresponding to EF-hands loaded with ions Ca^2+^, displays profiles mostly concentrated around 200 Å^2^, with some replica displaying few jumps up to 900 Å^2^. By contrast, the N-CaM patch show much more diversity spanning a range of 200–600 Å^2^. Similar trends have been observed for accessible surfaces of methionines in previous simulations of the literature (Yang et al., 2004), with a cumulative exposed surface of N-CaM Met of about 26 Å^2^ in apo-form and 88 Å^2^ with Calcium, whereas the C-CaM methionines displayed cumulative exposed surfaces of 45Å^2^ in apo-form and 124 Å^2^ with Calcium (Table 4 of Yang et al., 2004). The paradoxical behavior of the C-CaM patch which is blocked at a smaller accessible surface value than the N-CaM patch can be explained by the presence of the C_*A*_ region of EF which blocks the motions of C-CaM, as C-CaM is inserted between helical domain and C_*A*_. At the contrary, one side of N-CaM interacts with the helical domain, whereas the opposite side of N-CaM is free, which allows more mobility of the N-CaM patch. The variations of hydrophobic patches in the complex EF/CaM are not similar to those described for the isolated CaM. Indeed, in the isolated CaM, the variations of hydrophobic patches favor the CaM interaction with signaling peptides, but this aspect is not relevant in the case of EF/CaM interaction.

**Figure 6:**
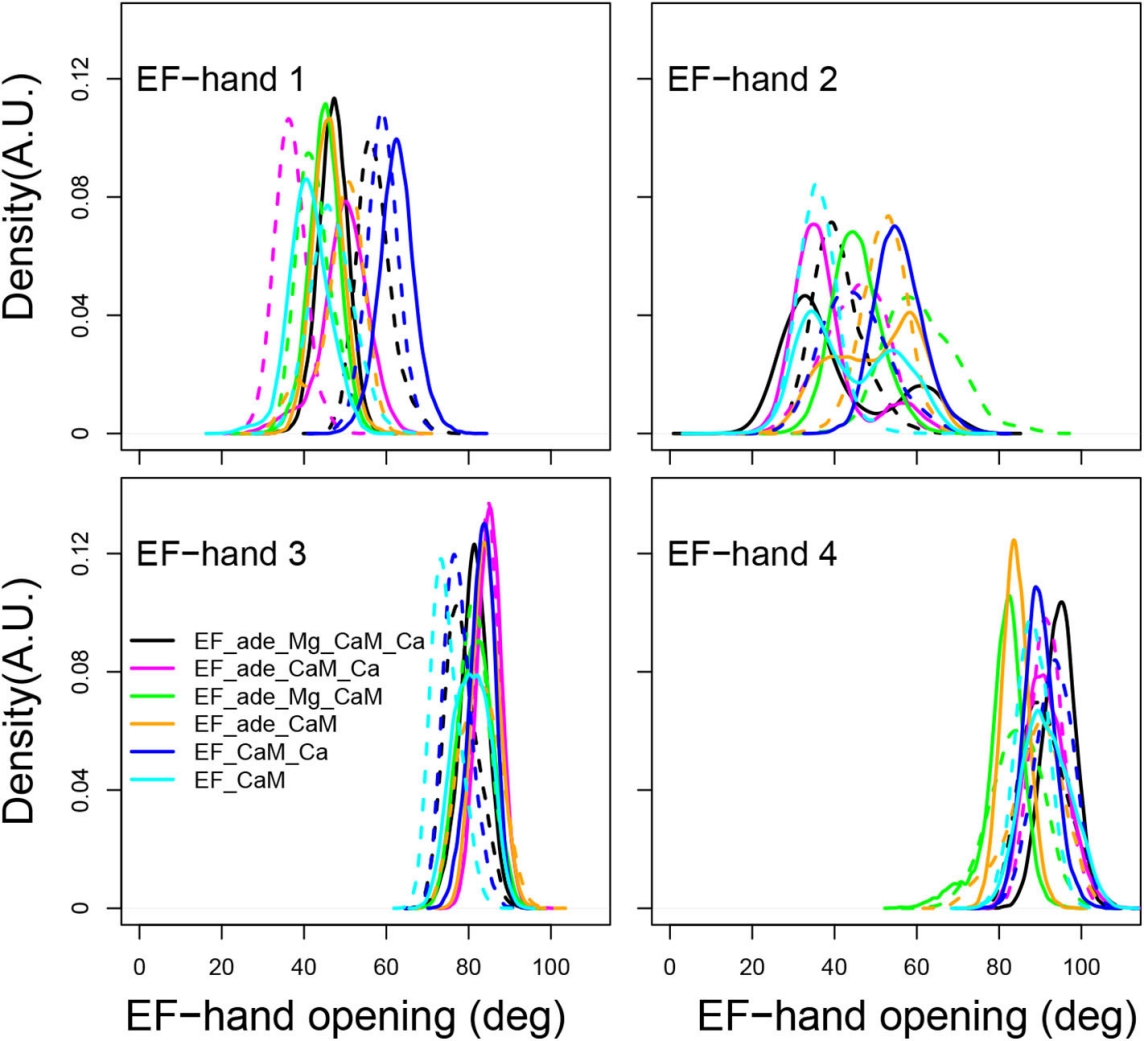
Angle of the EF-hands (deg) calculated between axes of α helices including residues 8-19 (helix I), 31-37 (helix II), 46-53 (helix III), 66-73 (helix IV), 83-92 (helix V), 103-110 (helix VI), 119-127 (helix VII), 139-145 (helix VIII). The angle of EF-hand 1 is the angle between helices I and II. The angle of EF-hand 2 is the angle between helices III and IV. The angle of EF-hand 3 is the angle between helices V and VI. The angle of EF-hand 4 is the angle between helices VII and VIII.

In isolated CaM, EF hands show a trend to open when CaM is loaded with calcium (Finn B.E. et al., 1995; Zhang et al., 1995). Similarly, in previous MD simulations of the EF/CaM complex (Laine et al., 2008), the C-CaM EF-hands, loaded with Ca^2+^, display more open α helices than the N-CaM EF-hands. Such behavior is also observed in the present simulations (Figure 6). In the present work, the EF-hands 3 and 4, located in C-CaM loaded with Ca^2+^ ions, display quite stable values around, respectively 80 and 90°, corresponding to open conformations. The EF-hand 4 fluctuates slightly more than the EF-hand 3, specially for some trajectories in which Calcium or Magnesium ions are absent (trajectories EF_ade_CaM and EF_ade_Mg_CaM). By contrast, the angles of EF-hands 1 and 2, located in N-CaM, display much wider variations among trajectories. The angle of EF-hand 1 is located in the 40–80° range for most of the trajectories, corresponding to conformations of the hand oscillating between closed and semi-open configurations. The angle of EF-hand 2 display the largest variations in the range 20–80°. For EF_ade_Mg_CaM trajectories (green curves), the EF-hand 2 explores open conformations with angles larger than 80° and, for one replica of EF_ade_Mg_CaM_Ca (black curves), EF_ade_CaM (yellow curves), and EF_CaM (cyan curves), displays equilibrium between closed and semi-open conformations. These EF-hands are located in the N-CaM lobe, which is not loaded with Calcium ions and interacts also in a much less intricate way with EF as only one side of N-CaM interacts with EF helical domain. For these two reasons, the angles between the two α helices are much more variable between the different conditions of simulations as well as between replicas of a given condition. In addition, the EF-hand 2 shows greater variability than the EF-hand 1, because it is farther away from the helical domain.

The overview of the CaM conformations reveals that the EF hands of N-CaM, which are not loaded with calcium ions, show much more heterogeneity in conformations, and populate conformations corresponding to closed and semi-open EF hand configurations. Otherwise, the overall conformations of CaM as well as the coordination of calcium are not strikingly modified among the trajectories recorded here.

### A Network of Amino-Acid Interactions Connect the EF Catalytic Site With CaM

**Figure 7:**
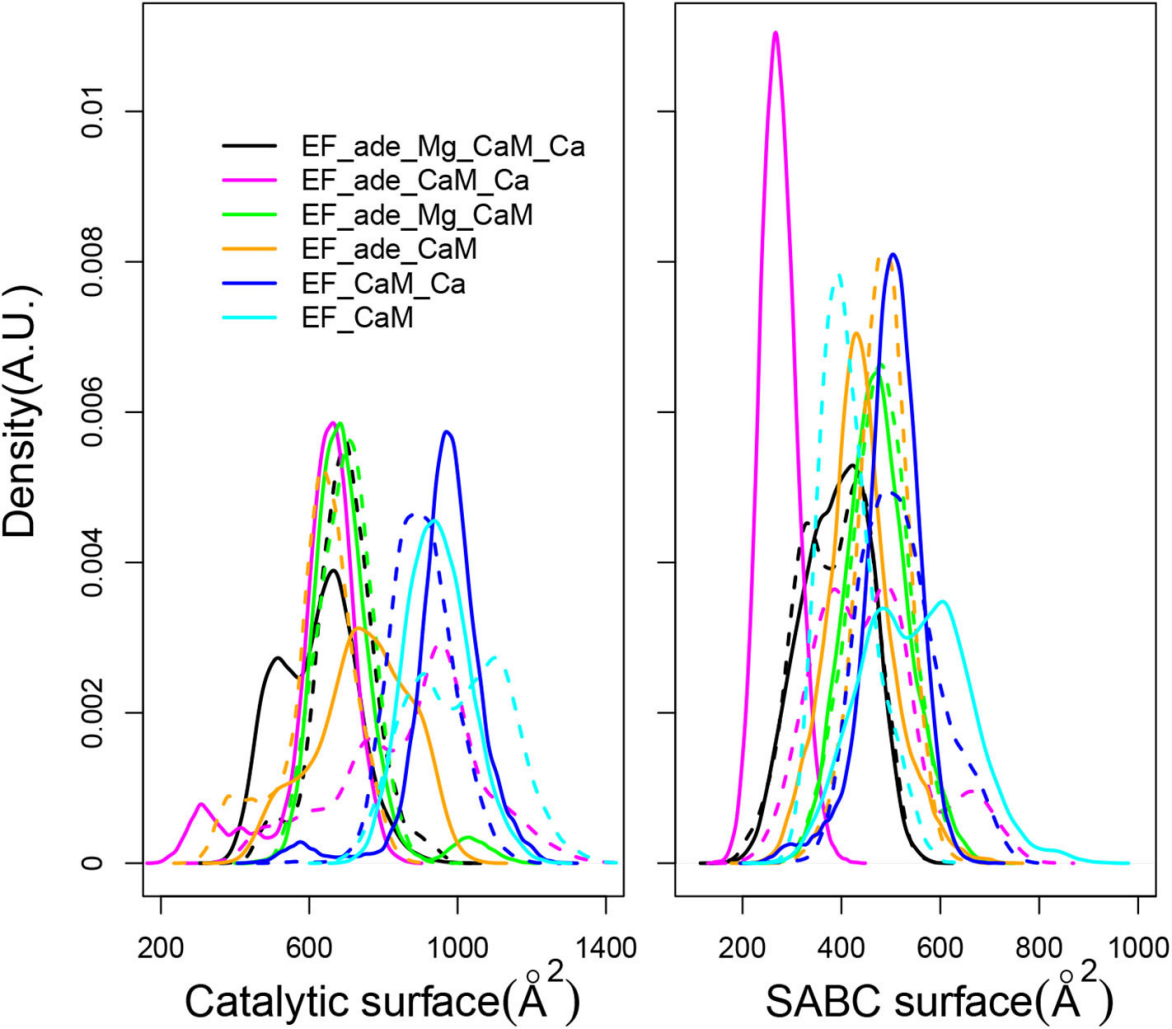
**(Left)** Distribution of accessible catalytic surface (Å^2^) of EF, calculated as the sum of the accessible surfaces of residues H351, K353, S354, K372, R329, K346, L348, D491, D493, H577, G578, T579, D582, N583, E588, F586, T548 of EF. **(Right)** Distribution of accessible surface (Å^2^) of the pocket SABC, calculated as the sum of the accessible surfaces of residues A496, P499, I538, E539, P542, S544, S550, W552, Q553, T579, Q581, L625, Y626, Y627, N629, and N709 of EF. For each trajectory, the plain and dashed curves correspond to replicas R1 and R2.

The EF activity has been investigated according to the accessible surface of the catalytic site, calculated as the sum of solvent accessible surfaces of residues H351, K353, S354, K372, R329, K346, L348, D491, D493, H577, G578, T579, D582, N583, E588, F586, and T548. This accessible surface varies in the 400–1,200 Å^2^ range (Figure 7, left). This range of values agrees with the average catalytic surfaces of EF previously observed in MD studies (Laine et al., 2008), as: 928 Å^2^ in the complex with 2 Calcium-loaded CaM, 866 Å^2^ in the complex with the 4 Calcium-loaded CaM and 501 Å^2^ in the complex with the apo CaM. Noticeably, in the presence of adefovir (black, yellow, magenta, and green curves) the surfaces are smaller around 400–800 Å^2^. This corresponds to a more closed catalytic site, in agreement with the inhibitory effect of adefovir. Removal of ion Mg^2+^ or removal of ions Ca^2+^ and Mg^2+^ in the presence of adefovir (magenta and yellow curves) induces a certain shift toward larger values, but the largest shifts toward the 800–1,200 Å^2^ range, is observed if adefovir is removed (cyan and blue curves). The trajectories EF_CaM_Ca (blue curves) corresponding to the activated EF display the most open catalytic site, which supports the use of accessible surface as an estimator of the EF catalytic activity.

The two following aspects concerning the interface between CaM and EF have been analyzed: (i) the accessible surface of the pocket SABC, (ii) the variation of interactions along a residue network spanning from the catalytic site to CaM.

The SABC pocket, previously used for the virtual screening having conducted to the discovery of thiophen ureoacids (Laine et al., 2010a), is formed by residues, A496, P499, I538, E539, P542, S544, S550, W552, Q553, T579, Q581, L625, Y626, Y627, N629, and N709, belonging to the three switches A, B, and C (Drum et al., 2002). The accessible surface of the SABC pocket (Figure 7, right) varies in a much smaller range of 200–800 Å^2^ than the catalytic pocket. Although the two pockets are defined by a similar number of residues, large differences are nonetheless observed for pocket SABC, sign of significant reorganizations in this region, resulting in some cases in the disappearance of a large accessible surface.

Initial analysis of the X-ray crystallographic structure 1PK0 (Shen et al., 2004b) has revealed a network of hydrogen bonds connecting adefovir and residues from catalytic site with EF residues at the EF/CaM interface and residues from CaM (Figure 1B). This network displays hydrogen bond and stacking interactions which have been monitored along trajectories (Supplementary Table 2). The contacts involve the residues T519, T548, Q553, G578, D582, N583 starting from the catalytic site and expanding to CaM. These residues are located in the switches A (residues 502-551), B (residues 578-591), and C (residues 630-659) (Figure 1B). Interestingly, in the X-ray structures of EF (Drum et al., 2002), these switches undergo a major reorganization between the inactive state and the active state of EF.

Overall, the proportion of formed interactions strongly decreases as soon as ions are removed from the system. In particular, the interactions involving ion Mg^2+^ are strongly reduced. In the initial X-ray crystallographic structure (PDB entry: 1PK0), the ion Yb^3+^ is penta-coordinated by three atoms from adefovir (O1-EMA, P3-EMA, O2-EMA) and two atoms from EF (Nϵ2-H577, O-Y492). In the MD trajectories, only the contact between Mg^2+^ and Nϵ2-H577 is stable along the trajectories EF_ade_CaM_Ca and EF_ade_Mg_CaM (Supplementary Table 2) and only three contacts are still present in the trajectory EF_ade_Mg_CaM_Ca: the ones with O2-ade and Nϵ2-H577 at a significant level and the one with O-Y492 at a negligible frequency. This loss of contacts could be due to the reduction of the charge and of the van der Waals radius between ions Yb^3+^ and Mg^2+^.

Among the interactions between adefovir and protein present in the structure 1PK0, only three (hydrogen bonds ade-H6/O-T548, ade-H7/O-T579, and stacking ade/N583) are still present along the trajectory EF_ade_Mg_CaM_Ca. Among them, the stacking between the indole part of adefovir and the aliphatic part of N583 is the only interaction significantly present along all trajectories (Supplementary Table 2). Nevertheless, as soon as the ions are removed from the system, the interaction frequency is reduced in one replica of EF_ade_Mg_CaM (B), EF_ade_CaM_Ca (C), and in all replicas of EF_ade_CaM (D). The adefovir hydrogen bonds conserved with protein mainly involve protons of the amine groups on the indole part. The contacts involving the phosphate group P1 in the structure 1PK0 are completely lost along all trajectories. This destabilization of the adefovir/protein contact and Magnesium contacts as soon as the ion Yb^3+^ is replaced by the ion Mg^2+^ could support an artifactual character of the structure 1PK0, in which the stability of adefovir in the catalytic site was obtained by the presence of the non-biological ion Yb^3+^.

The hydrogen bond and stacking interactions involving EF and CaM residues are reduced between trajectories EF_ade_Mg_CaM_Ca (A) and EF_ade_Mg_CaM (B) and between trajectories EF_ade_CaM_Ca (C) and EF_ade_CaM (D), when the ions Ca^2+^ are removed from the system. Overall, the removal of Ca^2+^ ions has more influence to reduce the contact stability as the removal of Mg^2+^. Indeed, six interactions decrease under a formation percentage of 10% if Ca^2+^ ions are removed, whereas no interaction decreases below this percentage in the absence of Mg^2+^. Interestingly, this influence is visible for hydrogen bonds established between the side chain guanidino group of R630 from EF and the side chain carboxyl groups of residues E84 and E87 from CaM, for the hydrogen bond between the sidechains of R540 from EF and the carboxyl groups of E87 from CaM, and also for the stacking between F628 from EF and R90 from CaM. Noticeably, since the CaM residues E84, E87, and R90 are located at the C terminal part of the α helix V, just before the EF-hand 3 in C-CaM, the presence or absence of Ca^2+^ ions in this lobe has a direct effect on the interaction EF/CaM.

Analysis of the EF/CaM interface, as well as the interactions within the catalytic site, highlight the influence of the variation in the composition of the system on the accessible surface of the catalytic site, impacting the enzymatic activity. In addition, a network of interactions between EF and CaM residues detected in the initial X-ray crystallographic structure, undergo strong destabilization when co-factors are removed: this network could be considered as a plausible communication path for allosteric correlation between CaM and the EF catalytic site.

### Analysis of Cavities Deformation to Detect Allosteric Pockets

In this section, we investigate the possibility to use the cavity tracking implemented in the software *mkgridXf* (Monet et al., 2019) along EF MD trajectories in order to predict allosteric sites in the EF/CaM complex. This approach is motivated by the following reasons. As already quoted in the introduction, several approaches for prediction of allosteric sites are based on a measure of the deformation of the protein described by an elastic network (Panjkovich and Daura, 2014; Guarnera and Berezovsky, 2016) or described by a normal mode perturbation (Greener and Sternberg, 2015). Also, a recent analysis of a large set of protein structures containing ligands showed (Alfayate et al., 2019) that the binding sites of allosteric ligands display larger deformations. Similarly, several bioinformatics approaches predict allosteric pockets as the ones on which ligand binding induces the largest variations in protein structures (Panjkovich and Daura, 2014; Guarnera and Berezovsky, 2019). Allostery has been thus repeatedly associated to larger local or global deformability. Beside, cavity tracking of *mkgridXf* (Monet et al., 2019) along MD trajectories made possible to correlate deformations of individual cavities to the principal components of the proteins global motions (Desdouits et al., 2015). The association of the observation made by Desdouits et al. (2015) with the literature approaches conducted us to investigate in the present work on the reliability of analyzing the cavity deformation to predict allosteric sites.

A systematic analysis and tracking of the cavities present in the complex was performed along trajectories using *mkgridXf* (Desdouits et al., 2015; Monet et al., 2019). The cavities were determined by rolling probes as described in section 2. Each cavity was tracked along MD trajectories using a description based on a consensus list of protein atoms delineating the cavities (Monet et al., 2019).

From each trajectory, only proteins EF and CaM have been kept, water molecules, ions and adefovir being removed. One frame every 40 was kept over the time interval 120–200ns of the trajectories EF_ade_CaM_Ca, EF_ade_CaM, EF_ade_Mg_CaM_Ca, EF_ade_Mg_CaM, EF_CaM_Ca and EF_CaM, and then concatenated. The two trajectory replicas series were then analyzed independently with *mkgridXf* in order to probe the reproducibility of the cavity analyses.

**Figure 8:**
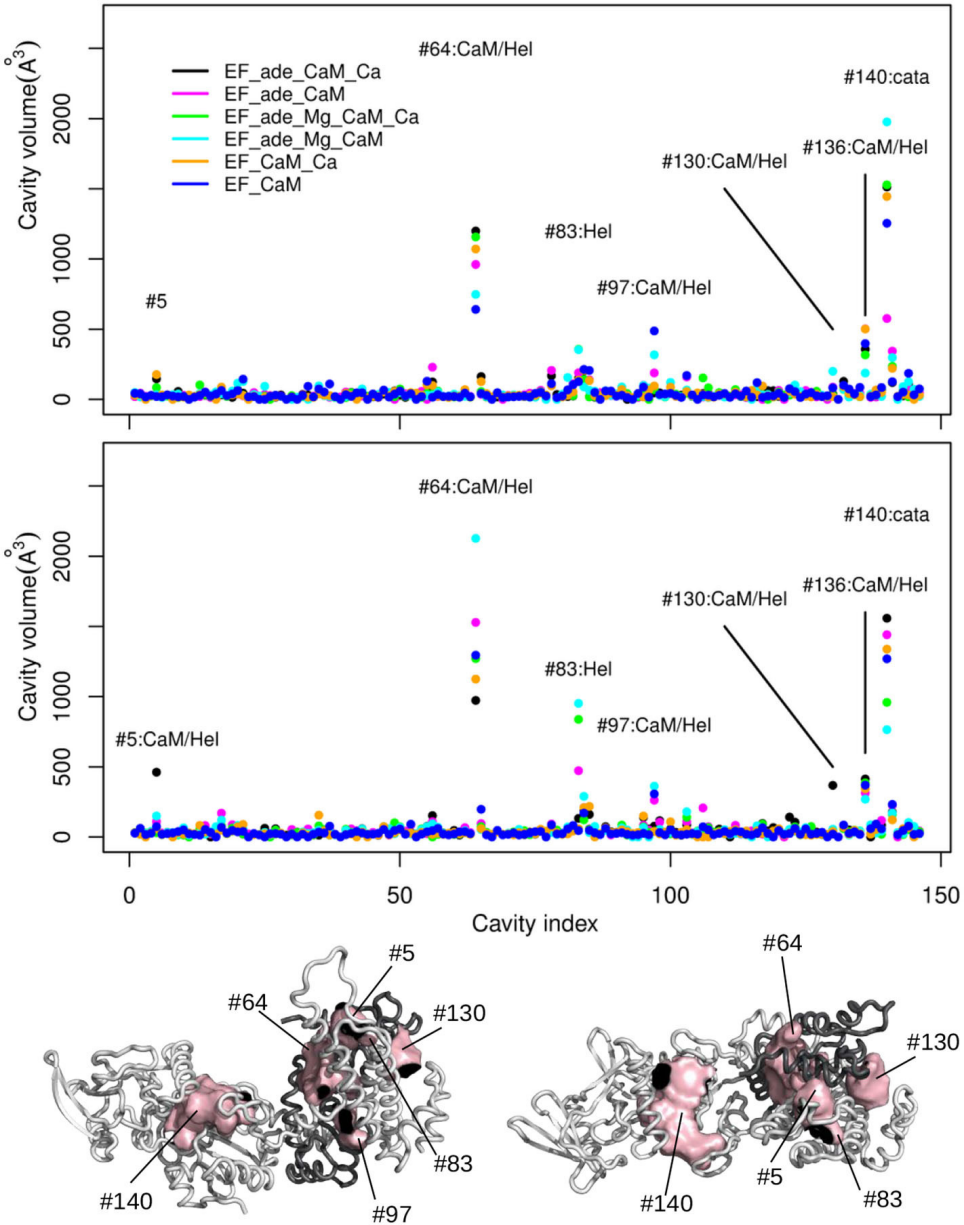
**(Top)** Averaged volumes (Å^3^) of the cavities detected by *mkgridXf* plotted along the cavity index. The two plots correspond to the two replicas series of the trajectories. The points are colored according to the trajectory on which the volume was averaged: EF_ade_CaM_Ca (black), EF_ade_CaM (magenta), EF_ade_Mg_CaM_Ca (green), EF_ade_Mg_CaM (cyan), EF_CaM_Ca (orange), and EF_CaM (blue). The cavities for which at least one volume larger than 250 Å^3^ has been observed, are labeled with the cavity number and annotated according to the cavity location: Hel, helical domain; cata, catalytic site; Hel/CaM, interaction interface between helical domain and CaM. **(Bottom)** Opposite views of the complex EF/CaM with CaM colored in dark gray. The cavities labeled on the plots are drawn in surfaces and colored in pink. CaM is colored in dark gray.

The deformations of protein cavities have been monitored through the variations of their volumes. The volumes of each cavity averaged along each trajectory are plotted as points along the cavity index, the points being colored according to the trajectory (Figure 8). For most of the cavities, the volumes are smaller than 100Å^3^ for all trajectories. Few of them (indexes #5, #64, #83, #97, #130, #136, #140) display larger volumes up to 2,500Å^3^ as well as large variations among the various trajectory conditions. The consensus residues defining each of these cavities have been determined using a cutoff of 2.5 on the residue score and are listed in Supplementary Table 3.

For both replicas, the cavity #140, located inside the catalytic site, is among the most variable cavities. Other very variable cavities are located at the interface between different CaM and EF regions. The CaM regions are the EF-hand 1 (#5, #130), the EF-hand 3 (#136), the EF-hand 4 (#97), and the central α-helix (#64). The EF regions are the helical region (#5, #64, #83, #97, #130) and the region C^*A*^ (#136). Two cavities located at the interface between the EF-hands and the region C^*A*^ (#97 and #136) display smaller volumes. According to the rationale exposed at the beginning of the section and which we explore on the EF/CaM complex, the “variable cavities” cited above and located at the interface between CaM and different EF regions are allosteric cavity candidates.

**Figure 9:**
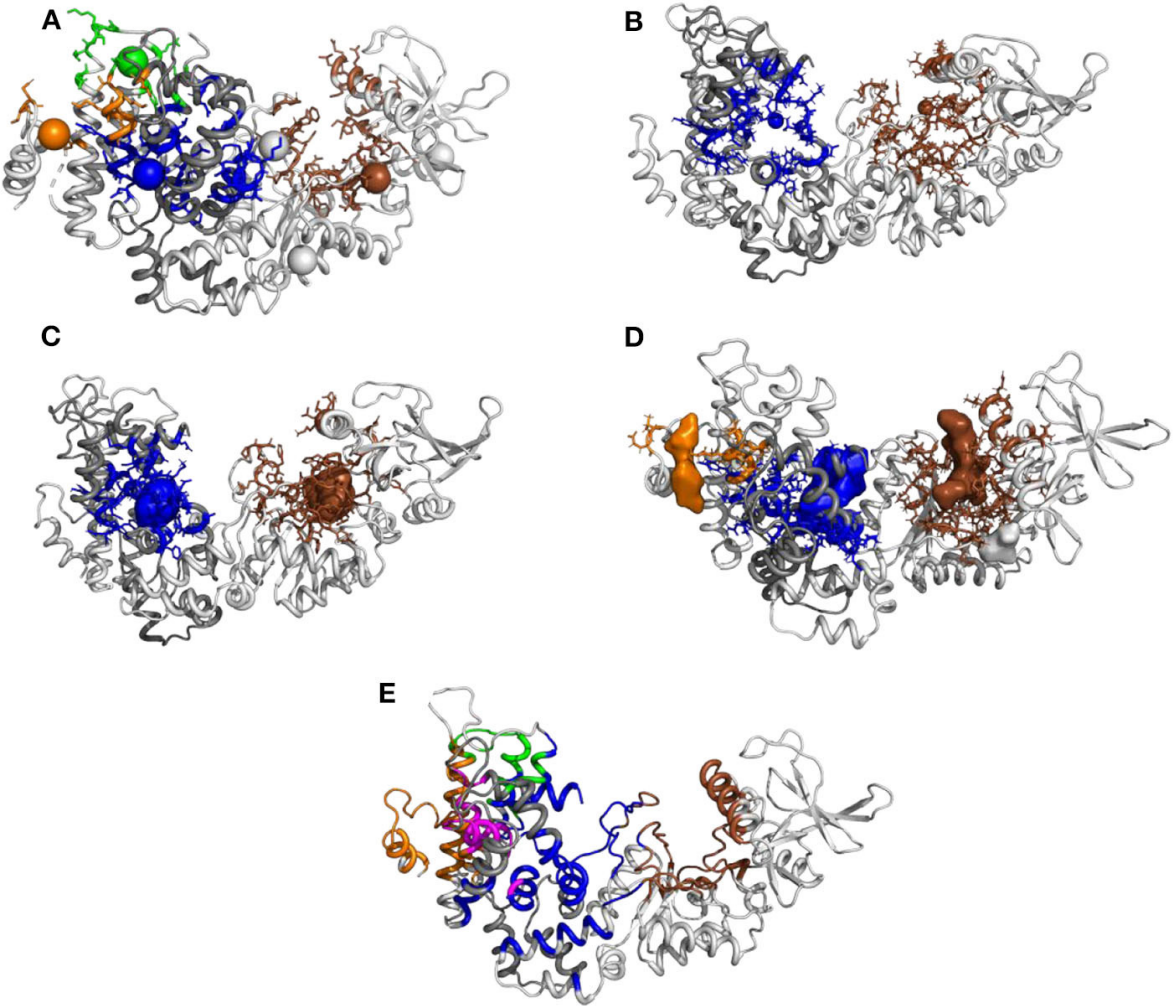
Prediction of pockets druggability or allostery on the initial EF/CaM structure 1PK0 using several methods: **(A)** PARS (bioinf.uab.cat/cgi-bin/pars-cgi/pars.pl), prediction of the druggable pockets, three of them correspond to *mkgridXf* cavities: 64 (blue), 130 (orange), and 140 (brown). **(B)** Deepsite (www.playmolecule.com/deepsite), prediction of two druggable pockets, displaying scores of 0.999 and corresponding to *mkgridXf* cavities: 64 (blue) and 140 (brown). **(C)** FTMap (ftsite.bu.edu), prediction of two druggable pockets, corresponding to *mkgridXf* cavities: 64 (blue) and 140 (brown). **(D)** POCASA (altair.sci.hokudai.ac.jp/g6/Research/POCASA_e.html), prediction of five druggable pockets, two of them corresponding to the *mkgridXf* cavity 140 (brown), and two others to the *mkgridXf* cavities: 64 (blue) and 130 (orange). **(E)** CavityPlus (www.pkumdl.cn:8000/cavityplus/index.php), prediction of the allosteric pockets influenced by the orthosteric pocket located in the catalytic site (brown). Four allosteric pockets are predicted with the corresponding scores: Cavity1 (3.36), Cavity5 (1.50), Cavity10 (0.75), Cavity4 (0.70). These cavities correspond to the *mkgridXf* cavities: 64 (blue), 130 (orange/magenta), 83 (green).

In order to probe this proposition, we compared the approach proposed here to current approaches in the literature. The initial conformation of the EF/CaM complex was thus processed with different web servers able to predict druggable or allosteric pockets (Figure 9). The servers PARS (Panjkovich and Daura, 2014), Deepsite (Jiménez et al., 2017), FTMap (Kozakov et al., 2015), POCASA (Yu et al., 2010), and CavityPlus (Xu et al., 2018) were used. Globally, the *mkgridXf* cavities 140 and 64, located respectively in the catalytic site and at the EF/CaM interface, correspond to predicted druggable pockets. The cavity 130, located in the helical domain, is present in some of the predicted sets of druggable pockets. Then, the server CavityPlus allows to identify the location of allosteric sites, using the CorrSite method (Ma et al., 2016). Using the catalytic site as the orthosteric site, the corresponding allosteric pockets predicted by CavityPlus correspond to the *mkgridXf* cavities 64, 130, and 83, located at the EF/CaM interface and in the helical domain of EF. To summarize, the set of *mkgridXf* cavities with large volume variation contain druggable pockets, and three of the *mkgridXf* cavities are predicted to be allosteric pockets with respect to the catalytic site. This last result supports the existence of an allosteric communication between the EF/CaM interface and the catalytic site.

**Figure 10:**
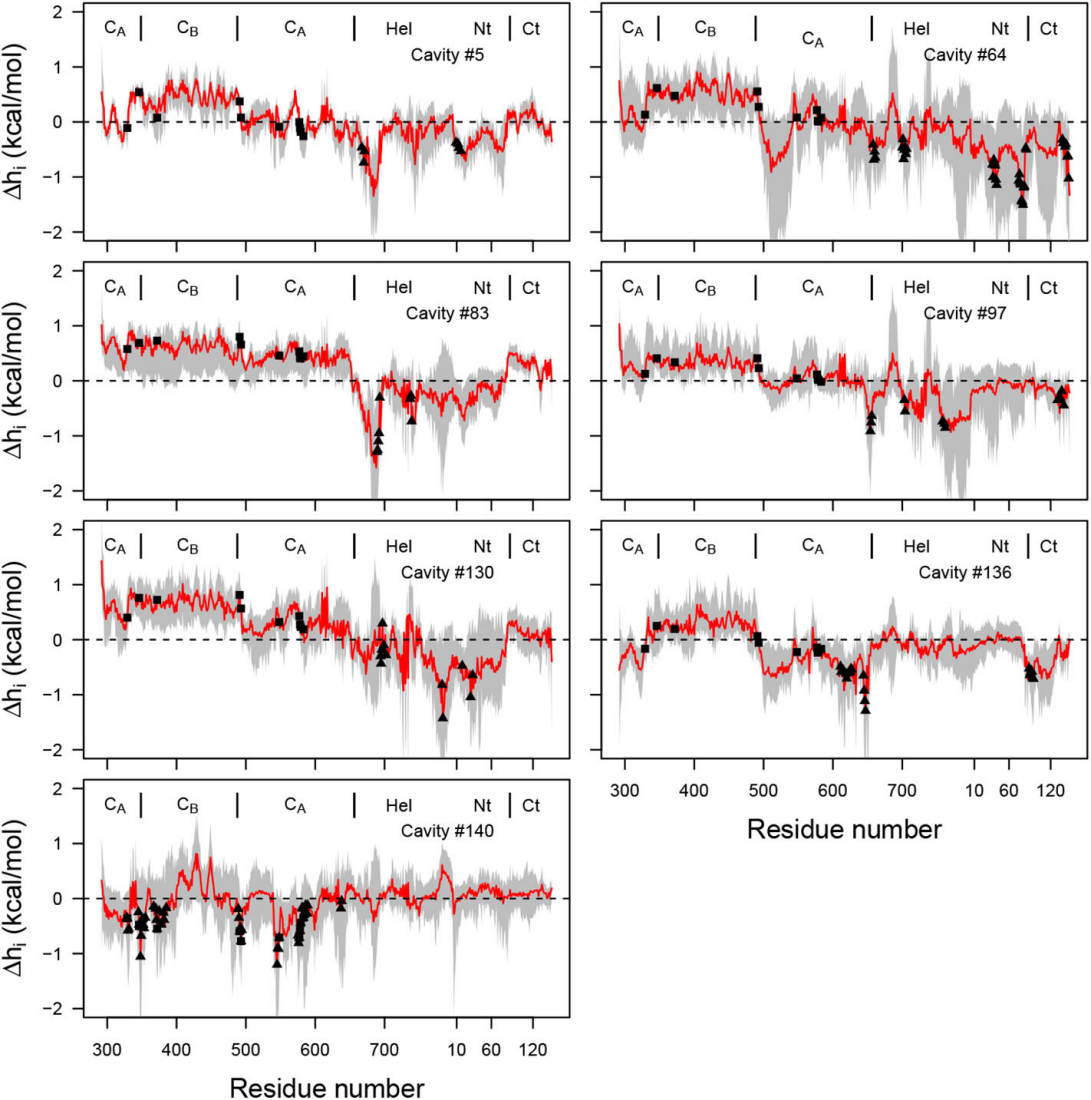
Plot of free energy $\Delta h_{i}^{\left(m↑\right)}$ (kcal/mol) profile, where the probe residue *m* samples the residues defining each of the selected cavities (Supplementary Table 3), and *i* samples all residues of the complex (x-axis). The average free energy profile is plotted in red with range of values drawn in gray. The residues defining the cavity (Supplementary Table 3) are shown with filled triangles, and the residues 329, 346, 372, 577, 491, 493, 548, 578, 579, 583 of the catalytic site are shown with filled rectangles. The dashed line corresponds to the zero energy value.

As described in the introduction, a theoretical frame for detection of allostery has been developed during the last 5 years: the structure-based statistical mechanical model of allostery (SBSMMA) (Guarnera and Berezovsky, 2019), based on a description of the protein as an elastic network between Cα. The effect of ligand is implicitly modeled through an additional energetic term added to the harmonic energy of the elastic network. The initial EF/CaM complex conformation has been processed on the AlloSigMA server (allosigma.bii.a-star.edu.sg/home) (Tan et al., 2020) implementing the SBSMMA model. The $\Delta h_{i}^{\left(m↑\right)}$ energy values, describing the allosteric communication between protein residues have been plotted for all residues involved in the *mkgridXf* cavities 5, 64, 83, 97, 130, 136, and 140 (Figure 10). The probe residues sample the cavity residues (filled triangles) and, for all cavities except 140, positive $\Delta h_{i}^{\left(m↑\right)}$ values describing a free energy change are observed for several residues located in the catalytic site (filled rectangle). The AlloSigMA results thus agree with an allosteric communication between *mkgridXf* cavities 5, 64, 83, 97, 130, 136, and the catalytic site.

The allosteric communication demonstrated by the variability of cavity volumes as well as by the energetics of the elastic network, is also supported by the variation of the network of interactions described in the previous section between the catalytic site and the helix V of CaM. This network is a plausible candidate for a communication path within the EF/CaM structure.

The cavities present in the EF/CaM complex were tracked along MD trajectories recorded with various perturbation conditions related to functional aspects of EF activity. In that way, the EF/CaM interface has been pointed out as a region containing pockets allosteric with respect to the orthosteric catalytic site. The targeting of these pockets by virtual screening has higher chances to conduct to the discovery of allosteric ligands.

## Discussion

In the present work, MD trajectories were recorded from the 1PK0 crystallographic structure of the EF/CaM complex, in the presence of various sets of co-factors: ions Ca^2+^ and Mg^2+^ and ligand adefovir.

The main finding from the comparison of trajectories is that the removal of ions has a strong effect on the conformations of the complex, at local and global levels. Indeed, the removal of Mg^2+^ ion destabilizes the interactions between EF and adefovir, but also affects contacts between EF and CaM. Similarly, the removal of Ca^2+^ ions destabilizes the interaction of EF with CaM, but also the geometry of the catalytic site and the EF/adefovir interactions even in the presence of ion Mg^2+^. This distant influence of ions agrees with the existence of a network of hydrogen bonds and stacking interactions which connects the catalytic pocket with the EF/CaM interface and which is destabilized if co-factors are removed. Noticeably a similar network of hydrogen bonds has been observed and validated using MD and mutagenesis (Selwa et al., 2014) in the adenylyl cyclase (AC) toxin from *Bordetella pertussis*.

Another observation is that the replacement of the ion Yb^3+^, observed in the initial X-ray crystallographic structure 1PK0 (Shen et al., 2004b), by the more biologically relevant Mg^2+^ ion induces a destabilization of numerous contacts between adefovir, ion and residues of the catalytic pocket. Consequently, the establishment of interactions due to the presence of Yb^3+^ ion could have enforced the binding of adefovir inhibitor to the catalytic site or induced the specific conformation of adefovir in the site. Previous computational analyses (Martínez et al., 2009, 2011) already highlighted the artifactual character of some ions observed in the catalytic site of various EF X-ray crystallographic structures.

The analysis of CaM conformations in the EF/CaM revealed that the removal of ions Ca^2+^ induces an unfitting of the conformation of CaM to its position in the complex as it is visible by the CaM central helix bending. The angles of the EF hands also show greater variations in N-CaM than in C-CaM, which could be related to a weaker interaction between EF and N-CaM.

The tracking of *mkgridXf* cavities in the EF/CaM complex along MD trajectories revealed large variations of cavity volumes in two regions: (i) the catalytic site and (ii) the interface between EF and CaM. The analysis of initial EF/CaM complex structure in the frame of the model SBSMMA (Guarnera and Berezovsky, 2019) has shown that the residues located in the *mkgridXf* cavities at the interface between EF and CaM, display allosteric communication with residues in the catalytic site. The procedure followed in the present manuscript has several points in common with the structure-based statistical mechanical model of allostery (SBSMMA). Indeed, SBSMMA states that the allostery can be quantitatively demonstrated using a model of elastic network which is deformed when the protein undergoes transition between unperturbed and perturbed states. In the present work, the unperturbed state is the MD trajectories recorded on the EF/CaM complex in presence of all co-factors, and the perturbed states are the MD trajectories recorded when one or more co-factors have been removed. In the frame of SBSMMA, the deformable cavities located at the interface between CaM and EF qualify them as being related in an allosteric way to the catalytic site and thus to the catalytic function of EF. Consequently, ligands designed to bind such cavities could have an allosteric effect on the catalytic activity of EF. In that respect, one should note that an inhibitor of EF, the compound 10506-2A, has been claimed to bind to the helical region (Lee et al., 2004).

During the last decade, many approaches have been developed for detecting pockets susceptible to bind allosteric ligands, and allosteric paths through protein structures (Daily and Gray, 2009; Mitternacht and Berezovsky, 2011; Bowman and Geissler, 2012; Panjkovich and Daura, 2014; Greener and Sternberg, 2015; Clarke et al., 2016; Guarnera and Berezovsky, 2016; Pfleger et al., 2017; Song et al., 2017; Huang et al., 2018; Abrusan and Marsh, 2019). These approaches are mostly based on a graph description of protein structures. The graphs are then analyzed either from the point of view of protein rigidity and graph theory, or from a more physical point of view of normal mode or elastic network analysis. In the present analysis, we decided to focus on the protein cavities. The relationship found here between the variability of *mkgridXf* cavity volumes and protein long distance communication is not surprising since such correlation has been observed previously (Desdouits et al., 2015) between *mkgridXf* cavities deformation and protein functional motions.

The proposition of using the volume variability of cavities for detection of allosteric communication has been only used here on the EF/CaM system. It is obvious that the application on a unique system does not constitute a general proof of concept. Nevertheless, use of cavities tracking along MD trajectories presents some advantages with respect to methods based on the modeling of protein via an elastic interaction network (Panjkovich and Daura, 2014; Guarnera and Berezovsky, 2016). Indeed, by contrast with the network where only one atom (generally Cα) per residues is included in the calculation, the cavity calculation and tracking take information about all atoms into account, as well as their mutual interactions and their interaction with the solvent and co-factors. Moreover, the model for internal dynamics of the complex is more realistic than the quadratic energy surface of the elastic network model. Finally, perturbing the system by removing co-factors, as ions, highly involved in the EF function, makes the observation of protein deformation more specifically related to the inhibition of EF function. However, all these improvements are accessible at the cost of a very intense computational effort.

## Data Availability Statement

The raw data supporting the conclusions of this article will be made available by the authors, without undue reservation.

## Author Contributions

IP, PG, AB, and TM designed the study. PG, AB, and TM wrote the manuscript. IP, DM, and TM recorded the trajectories and performed the analyses. All authors contributed to the article and approved the submitted version.

## Conflict of Interest

The authors declare that the research was conducted in the absence of any commercial or financial relationships that could be construed as a potential conflict of interest.
