# Supplementary material for: Analyzing In Silico the Relationship Between the Activation of the Edema Factor and Its Interaction With Calmodulin
Source: Front Mol Biosci. 2020 Dec 4;7:586544. doi: 10.3389/fmolb.2020.586544 (PMC7746812; doi:10.3389/fmolb.2020.586544)
Supplement: Supplementary file 1 [file Data_Sheet_1.pdf]

November 19, 2020

Supplementary information: analyzing *in silico* the relationship between the activation of the edema factor and its interaction with calmodulin

Irène Pitard[1,2,3], Damien Monet[1,2,3], Pierre L. Goossens[4], Arnaud Blondel[1,2] and Thérèse E Malliavin[1,2]

[1] Institut Pasteur and CNRS UMR 3528, rue du Dr Roux, Unité de Bioinformatique Structurale, 75015 Paris, France

[2] Institut Pasteur and CNRS USR 3756, rue du Dr Roux, Centre de Bioinformatique, Biostatistique et Biologie Intégrative, 75015 Paris, France

[3] Ecole Doctorale Université Paris Sorbonne, 75005 Paris, France

[4] Institut Pasteur, rue du Dr Roux, Unité Yersinia, 75015 Paris, France

Key words: protein-protein interaction; *Bacillus anthracis*; virulence factor; cavity detection; allostery

# Corresponding author

Thérèse E Malliavin

Institut Pasteur and CNRS UMR 3528, Unité de Bioinformatique Structurale,

25 rue du Dr Roux, 75015 Paris, France

E-mail address: therese.malliavin@pasteur.fr

Methods

Self-organizing maps

A clustering approach, the **S**elf-**O**rganizing **M**aps (SOM), which is an artificial neural network (ANN) trained using unsupervised learning, was used to extract representative conformations from the molecular dynamics (MD) trajectories [1]. The conformations sampled along trajectories were encoded from the distances d_{ij} calculated between the n C_α atoms of the complex EF/CaM, by diagonalizing the covariance matrix C:

$$C_{i,j} = \frac{1}{n} \sum_{k=1}^{n} \sum_{l=1}^{n} (d_{i,k} - \bar{d}_i)(d_{l,j} - \bar{d}_j) \tag{1}$$

where $\bar{d}_i = \frac{1}{n} \sum_{j=1}^{n} d_{i,j}$. The information contained in the matrix C can be equivalently represented by its four largest eigenvalues along with the corresponding eigenvectors. The eigenvalue and eigenvector descriptors are used to train a periodic Euclidean 2D self-organizing map (SOM), defined by a three-dimensional matrix.

The self-organizing maps were initialized with a random uniform distribution covering the

range of values of the input vectors. At each step, an input vector is presented to the map, and the neuron closest to this input is updated. The maps are trained in two phases. During the first phase, the input vectors are presented to the SOM in random order to avoid mapping bias with a learning parameter of 0.5, and a radius parameter of 36. During the second phase, the learning and radius constants are decreased exponentially from starting values 0.5 and 36, respectively, during 10 cycles of presentation of all the data in random order. Once the calculation of the SOM has been realized, the processed MD trajectory has been transformed into a map in which each pixel corresponds to a series of similar conformations of the complex EF/CaM. The conformations of the complex corresponding to local maxima of homogeneity in the SOM map, are further used as representative conformations of the MD trajectory.

References

[1] Bouvier G, Desdouits N, Ferber M, Blondel A, Nilges M. An automatic tool to analyze and cluster macromolecular conformations based on Self-Organizing Maps. *Bioinformatics* **31** (2014) 1–3.

Figure S1

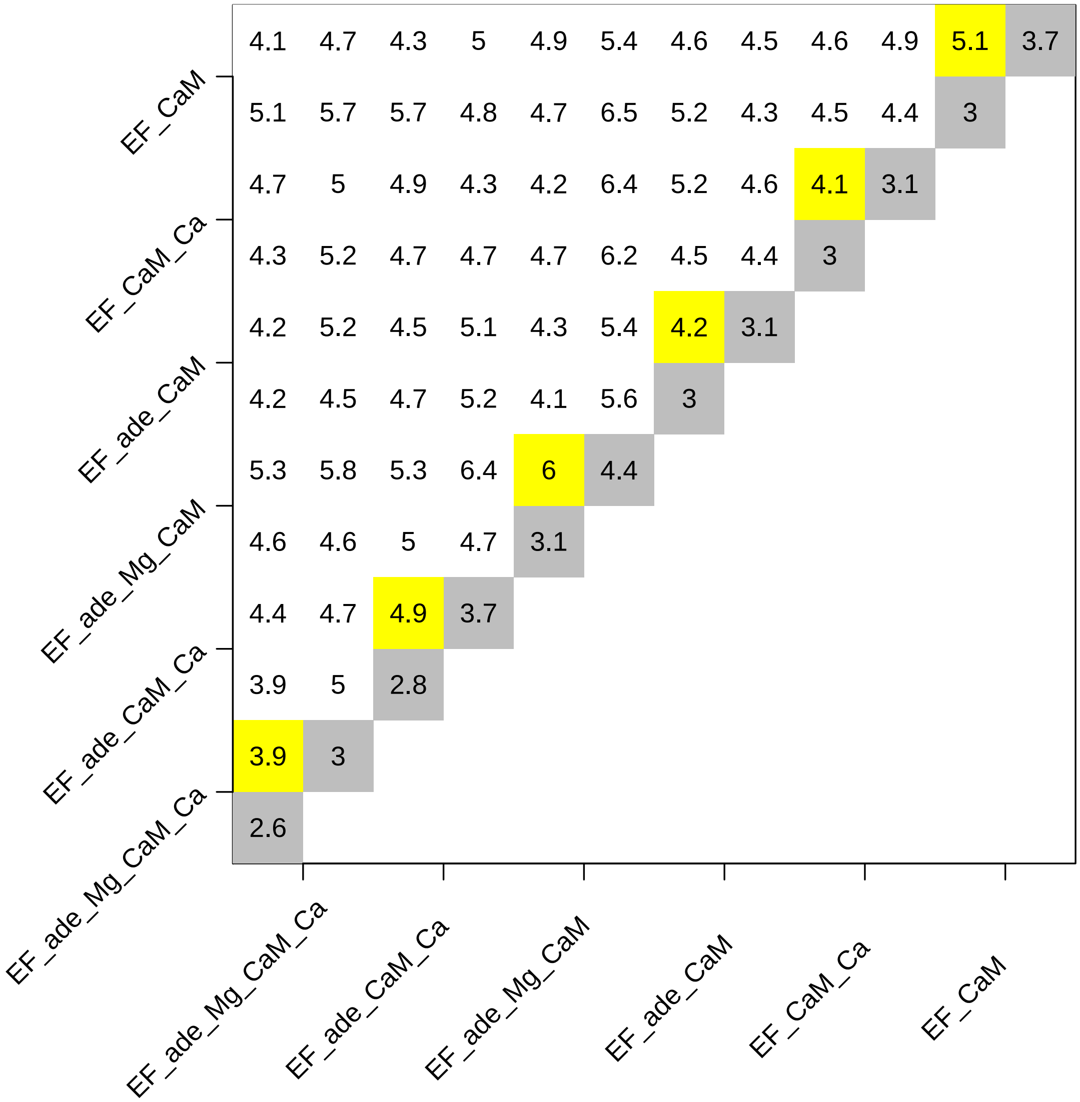

Figure S1: Average coordinate root mean square deviation (RMSD: Å) between representative conformations extracted from each trajectory replica using the self-organizing map (SOM) approach. The RMSD values were calculated by superimposing the heavy backbone atoms. The comparisons of the conformations within a given replica are in grey-colored cells. The comparisons of the conformations between two replicas of a given trajectory are in yellow-colored cells.

Calcium	CaM atom	EF_ade_Mg_CaM_Ca	EF_ade_CaM_Ca	EF_CaM_Ca
Cal1	O-Y^{99}	2.3 ± 0.1<br>2.3 ± 0.1	2.3 ± 0.1<br>2.3 ± 0.1	2.3 ± 0.1<br>2.3 ± 0.1
Cal1	$O\delta1$-D^{93}	3.2 ± 0.84<br>2.1 ± 0.05	2.1 ± 0.05<br>2.1 ± 0.05	2.1 ± 0.1<br>2.1 ± 0.05
Cal1	$O\delta2$-D^{93}	3.8 ± 0.2<br>2.7 ± 0.8	3.8 ± 0.2<br>3.8 ± 0.2	3.6 ± 0.2<br>3.6 ± 0.2
Cal1	$O\delta1$-D^{95}	3.7 ± 0.2<br>3.8 ± 0.2	3.7 ± 0.2<br>3.7 ± 0.2	2.2 ± 0.1<br>2.2 ± 0.1
Cal1	$O\delta2$-D^{95}	2.1 ± 0.05<br>2.1 ± 0.05	2.1 ± 0.05<br>2.1 ± 0.05	3.6 ± 0.1<br>3.6 ± 0.1
Cal1	$O\epsilon1$-E^{104}	2.2 ± 0.1<br>2.3 ± 0.1	2.2 ± 0.1<br>2.2 ± 0.1	2.2 ± 0.1<br>2.2 ± 0.1
Cal1	$O\epsilon2$-E^{104}	2.2 ± 0.1<br>2.2 ± 0.1	2.2 ± 0.1<br>2.2 ± 0.1	2.3 ± 0.1<br>2.3 ± 0.1
Cal2	O-Q^{135}	2.3 ± 0.1<br>2.3 ± 0.1	2.4 ± 0.1<br>2.4 ± 0.1	2.3 ± 0.1<br>2.3 ± 0.1
Cal2	$O\delta1$-D^{131}	2.1 ± 0.05<br>2.1 ± 0.05	2.1 ± 0.05<br>2.1 ± 0.05	2.1 ± 0.05<br>2.1 ± 0.05
Cal2	$O\delta2$-D^{131}	3.7 ± 0.2<br>3.6 ± 0.2	3.7 ± 0.2<br>3.7 ± 0.2	3.8 ± 0.2<br>3.8 ± 0.2
Cal2	$O\delta1$-D^{133}	2.2 ± 0.1<br>2.2 ± 0.1	2.2 ± 0.1<br>2.2 ± 0.1	2.1 ± 0.1<br>2.1 ± 0.05
Cal2	$O\delta2$-D^{133}	3.6 ± 0.1<br>3.6 ± 0.1	3.6 ± 0.1<br>3.6 ± 0.1	3.8 ± 0.2<br>3.8 ± 0.2
Cal2	$O\epsilon1$-E^{140}	2.2 ± 0.1<br>2.2 ± 0.1	2.2 ± 0.1<br>2.2 ± 0.1	2.2 ± 0.1<br>2.2 ± 0.1
Cal2	$O\epsilon2$-E^{140}	2.3 ± 0.1<br>2.3 ± 0.1	2.3 ± 0.1<br>2.3 ± 0.1	2.3 ± 0.1<br>2.2 ± 0.1

Table S1: Average distances (Å) between Calcium ions (labeled Cal1 and Cal2) located in CaM EF-hands 3 and 4 and atoms of C-CaM residues. The two lines of each table cell correspond to the two replicas recorded for each trajectory.

interaction type	A	B	C	D	E	F
Ion Mg^{2+}						
MG/O2-ade	100/32	-	0.0/0.0	-	-	-
MG/O-Y492	3.5/3.5	-	0.0/0.0	-	-	-
MG/Nϵ2-H577	100/100	-	100/100	-	-	-
adefovir/protein	A	B	C	D	E	F
H14-ade/Oδ-D493	0.7/2.8	0/0	0/0	0/0	-	-
P1-ade/Hζ-K346	0/0	0/0	0/0	0/0	-	-
O5-ade/sidechain-R329	1.3/0	2.1/0	4.6/0.5	0.1/4.4	-	-
H13-ade/sidechain-R329	0/8.9	0/0	0/0	0/0	-	-
H11-ade/Oδ-D491	1.5/0	0/0	0/0	0/0	-	-
H6-ade/O-T548	92/96	0/0	0/0	0/0	-	-
H6-ade/HN-T548	26/5	0/0	0/0	0/0	-	-
H7-ade/O-G578	12/4	0/0	0/0	0/0	-	-
H7-ade/O-T579	79/95	0/0	0/0	0/0	-	-
N3-ade/O-T548	0/0	0/0	0/0	0/0	-	-
Stacking ade/N583	92/99	50/96	88/23	2.9/23	-	-
EF/EF	A	B	C	D	E	F
Hγ1-T579/Oδ-D582	99/100	45/94	100/34	51/81	83/11	23/83
Oϵ1-Q553/HN-T792	35/52	26/19	75/56	17/0	0.7/6	34/0
K346-Hζ1/G352-O	88/92	99/94	0/0	34/60	6/1.4	0.9/0.2
Q553-Hϵ/D792-Oδ	60/97	36/44	100/22	21/49	4/3.3	12/15
Q553-Hϵ/S550-Oγ	17/3.4	2/2.5	58/11	1.4/0.5	8/8	3.7/6
Stacking R540/Y627	58/17	0.2/1.7	91/23	9/0.1	13/12	2.2/69
H577-Hδ1/G578-O	93/88	94/93	28/11	38/33	41/24	29/25
N581-Oϵ1/N629-HN	99/99	95/99	92/98	90/99	93/80	85/99
D582-Oδ/Y627-HH	77/86	27/19	91/22	37/52	63/30	53/46
N583-O/N629-Hδ	36/29	82/39	28/58	35/68	38/90	27/58
Stacking I615/Y626	70/89	60/49	80/72	63/76	75/73	79/59
Stacking Y626/I619	0/0	0/0	0/0	0/0	0/0	0/0
EF/CaM	A	B	C	D	E	F
R630-sidechain/E87-Oϵ	97/73	24/8	69/59	60/16	53/19	37/100
R630-sidechain/E84-Oϵ	69/29	21/2.4	40/34	20/33	7/4	2.5/98
sidechain-R540/Oϵ-E87	68/19	3.4/25	92/48	4.5/0	21/0.5	1.8/70
Stacking F628/R90	49/13	2.3/7	52/32	7/11	8/5	8/86

Table S2: Van der Waals and hydrogen bonds interactions observed along MD trajectories connecting atoms from EF, CaM and adefovir (ade), as well as ions Ca^{2+} and Mg^{2+}. The selected interactions were picked up in the initial X-ray crystallographic structure 1PK0. The percentage of formation correspond to the percentage of frames for which the distance is smaller than 2.5 Å in the case of hydrogen bonds and smaller than 4.5 Å in the case of stacking interactions. The trajectory names given in the table correspond to: A=EF_ade_Mg_CaM_Ca, B=EF_ade_Mg_CaM, C=EF_ade_CaM_Ca, D=EF_ade_CaM, E=EF_CaM_Ca, F=EF_CaM.

Cavity number	Consensus residue list
5	EF-667/EF-670/EF-671/CaM-9/CaM-12/CaM-13/CaM-16
64	EF-652/EF-658/EF-659/EF-660/EF-661/EF-664/EF-700/EF-701/ EF-702/EF-703/EF-704/EF-705/EF-706/ CaM-7/CaM-10/CaM-11/CaM-36/CaM-37/CaM-38/CaM-39/ CaM-40/CaM-41/CaM-42/CaM-71/CaM-74/CaM-75/CaM-76/ CaM-77/CaM-78/CaM-79/CaM-80/CaM-81/CaM-82/CaM-84/ CaM-85/CaM-137/CaM-138/CaM-141/CaM-142/CaM-144/ CaM-145/CaM-146
83	EF-666/EF-670/EF-689/EF-690/EF-691/EF-692/EF-693/EF-734/ EF-737/EF-738/EF-739/EF-740/EF-744/EF-748
97	EF-654/EF-655/EF-656/EF-703/EF-704/EF-758/EF-761/EF-762/ CaM-98/CaM-130/CaM-132/CaM-134/CaM-136/CaM-139
130	EF-694/EF-695/EF-696/EF-697/EF-698/EF-699/EF-702/EF-726/ EF-783/EF-784/CaM-18/CaM-30/CaM-33
136	EF-500/EF-611/EF-612/EF-614/EF-615/EF-619/EF-620/EF-623/EF-624/ EF-625/EF-626/EF-627/EF-644/EF-645/EF-646/EF-647/ CaM-86/CaM-89/CaM-90/CaM-91/CaM-92/CaM-93/CaM-94/CaM-95
140	EF-328/EF-329/EF-330/EF-331/EF-345/EF-346/EF-347/EF-348/EF-349/ EF-350/EF-351/EF-352/EF-353/EF-355/EF-363/EF-367/EF-370/EF-371/ EF-372/EF-375/EF-377/EF-378/EF-381/EF-382/EF-385/EF-489/EF-490/ EF-491/EF-492/EF-493/EF-494/EF-545/EF-546/EF-547/EF-548/EF-575/ EF-576/EF-577/EF-578/EF-579/EF-581/EF-582/EF-583/EF-584/EF-585/ EF-586/EF-587/EF-588/EF-589/EF-637/EF-638

Table S3: List of cavities with large volume variability detected using *mkgridXf* along with the residue consensus definition of each cavity. The EF and CaM residues numbers are indicated by the name "EF and "CaM". Residues were detected using a cutoff of 2.5 on the score describing the belonging of the residue to the cavity.
